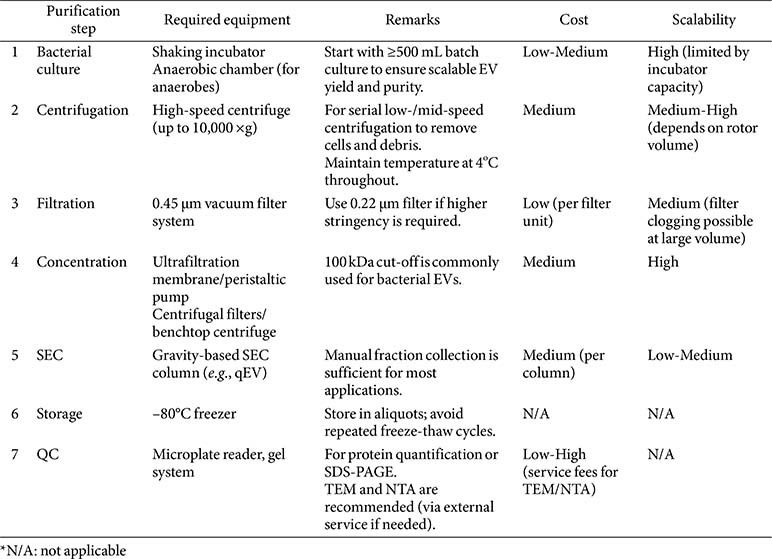# Standardizing Bacterial Extracellular Vesicle Purification: A Call for Consensus

**DOI:** 10.4014/jmb.2506.06011

**Published:** 2025-09-23

**Authors:** Dongsic Choi, Eun-Young Lee

**Affiliations:** 1 Department of Biochemistry, College of Medicine, Soonchunhyang University, Cheonan 31151, Republic of Korea; 2 Research Center for Extracellular Particles, College of Medicine, Soonchunhyang University, Cheonan 31151, Republic of Korea

**Keywords:** Extracellular vesicle, bacteria, outer membrane vesicle, microbiota, purification

## Abstract

Bacteria constitutively produce extracellular vesicles (EVs), which play a pivotal role in linking bacterial physiology to therapeutics. Despite significant advances over the past decade, critical challenges persist in isolating EVs due to the absence of standardized purification methods. While the International Society for Extracellular Vesicles provides well-defined minimal guidelines for human EVs, bacterial EV research still faces inconsistencies across laboratories, limiting reproducibility and broader utility. This review provides an in-depth overview of technical aspects of bacterial EV purification and gives future perspectives for achieving high-purity and reproducibility when isolating EVs from diverse bacterial species. It further addresses the necessity of distinguishing bacterial EVs from host-derived vesicles and potential contaminants, particularly in clinical and microbiota contexts. Establishing gold-standard protocols and defining *bona fide* bacterial EVs in parallel with the rigor seen in human EV studies will advance our understanding of their roles in host-microbe interactions, microbiota dysbiosis, and therapeutic potential.

## Introduction

In living organisms, almost all eukaryotic cells produce extracellular vesicles (EVs) for intercellular communication [1]. Bacteria also release EVs during their normal growth, characterized by nanoscale, membrane-encapsulated structures [2-4]. Although bacterial and eukaryotic EVs share some physicochemical properties (*e.g.*, size, density, charge), bacterial EVs exhibit unique heterogeneity and distinct structural features defined by size and composition closely related to their biogenesis pathways [3]. Unlike eukaryotic EVs enriched in sphingomyelin and canonical markers such as CD63 [5, 6], bacterial EVs display pathogen-associated molecular pattern (PAMP)-rich membranes and biogenesis-dependent cargo including proteins and nucleic acids [3, 7]. These differences in membrane composition, cargo, and biogenesis necessitate bacteria-specific purification and quality control (QC) parameters.

Historically, bacterial EV research has focused predominantly on Gram-negative bacteria known to produce outer membrane vesicles (OMVs), whereas their Gram-positive counterparts were long thought unable to generate EVs as part of their normal physiology [2]. However, the discovery and characterization of Gram-positive bacterial EVs, especially from *Staphylococcus aureus*, bridged critical gaps in our understanding of EV evolution and demonstrated the universality of EV production across bacterial species [8]. Studies on EV production have since been extended to other species, including mycobacteria [9], mycoplasma [10], and yeast [11], highlighting the natural shedding of EVs across microbes. Moreover, the widespread use of anaerobic chambers in laboratory settings has provided low-oxygen conditions that mimic the host gut environment, enabling the isolation of EVs from anaerobic bacteria, including commensal species such as *Akkermansia muciniphila* [12, 13] and various Bacteroidetes strains [14-16]. These advancements have broadened our understanding of bacterial EVs, particularly their emerging roles in human microbiota, host-microbe interactions, and their potential applications as biomarkers in diagnostics and therapeutics.

Despite advances in the field of bacterial EVs, comprehensive guidelines for their purification methods are still lacking. Bacterial EVs can vary in type, function, and molecular cargo depending on the species, strain, growth conditions, and biogenesis [17, 18]. Therefore, differentiating EV subtypes such as OMVs, cytoplasmic membrane vesicles (CMVs), vesicles with distinct biogenetic origins remains a technical challenge. Inconsistencies in purification protocols across laboratories can result in substantial differences of EV yield, purity, and function [19]. While Gram-negative and Gram-positive EVs originate from distinct biogenesis mechanisms, similar purification methods are often applied to both [20, 21]. Early studies on Gram-positive bacterial EVs have adapted protocols originally designed for Gram-negative OMVs, raising concerns about their suitability due to their differences in size, content, density, and other physicochemical properties [8, 22, 23]. These differences might also lead to selective loss of Gram-positive EV subpopulations when general protocols for Gram-negative bacteria or eukaryotes are applied [17]. To address these issues, optimizing growth phase, buffer composition, and centrifugal parameters should be considered to enhance Gram-positive EV yield and purity. Particularly, density gradient ultracentrifugation should be applied to reduce contaminants such as cell wall fragments and enrich high-purity EVs [8, 24]. Moreover, bacterial EV properties may vary within the same strain depending on growth conditions and stressors [25-27]. Therefore, establishing standardized yet strain-specific guidelines remains a high priority to optimize EV recovery, improve quality, and ensure reproducibility.

Unlike bacterial EVs, eukaryotic EV isolation is far more standardized with established minimal guidelines [1, 17, 28]. Notably, the International Society for Extracellular Vesicles (ISEV) has continuously refined its guidelines with the latest update, Minimal Information for Studies of Extracellular Vesicles (MISEV) 2023 [1], which provides comprehensive recommendations for EV research across various biological sources. These guidelines emphasize rigor, reproducibility, and transparency, addressing key challenges including nomenclature, separation methods, characterization techniques, and functional analyses [20]. ‘EV’ is an umbrella term encompassing all subtypes of eukaryotic vesicles including exosomes, ectosomes (microvesicle, microparticle), and apoptotic bodies, each of which displays distinct characteristics such as size, shape, density, and molecular markers based on biogenesis pathways [29-31]. Classically, EVs were isolated by differential centrifugation. However, this method cannot discriminate diverse EV subtypes. Current research studies on eukaryotic EVs have applied complementary techniques such as density gradient ultracentrifugation, size-exclusion chromatography, affinity chromatography, asymmetric flow field-flow fractionation, and others to discriminate the heterogenic nature of EVs, enabling extensive characterization of specific subtypes [1]. While no single marker or method can universally define all eukaryotic EVs, immunoaffinity-based isolation using specific markers can enrich particular subtypes [32-34]. In contrast, bacterial EV studies have yet to establish a detailed classification system for EV subtypes. For instance, Gram-positive EVs are broadly categorized as CMVs without further subclassification [7]. In addition, Gram-negative bacterial EVs are classified into OMVs, outer-inner membrane vesicles (OIMVs), and explosive outer membrane vesicles (EOMVs). However, their detailed molecular and biophysical profiles remain poorly defined [35-37]. Akin to those in eukaryotic EV research, it would be necessary to focus on comprehensive characterization of EV types by their biochemical composition, surface markers, genetic cargo, and physicochemical properties.

This review primarily focuses on purification methods for bacterial EVs, emphasizing *in vitro* culture-based isolation techniques. While it highlights challenges of isolating bacterial EVs from biological samples, where host-derived EVs are predominant, the primary goal is to establish a straightforward procedure for achieving high-purity and reproducibility of EV isolations from bacterial cultures. Effective methodologies will be pivotal in bacterial EV research for robust comparative analyses across strains. Ultimately, these approaches hold promise for unlocking the potential of EVs as reliable biomarkers and safe therapeutic tools in clinical settings.

## Optimization of Bacterial Culture Conditions

**Fig. 1:**
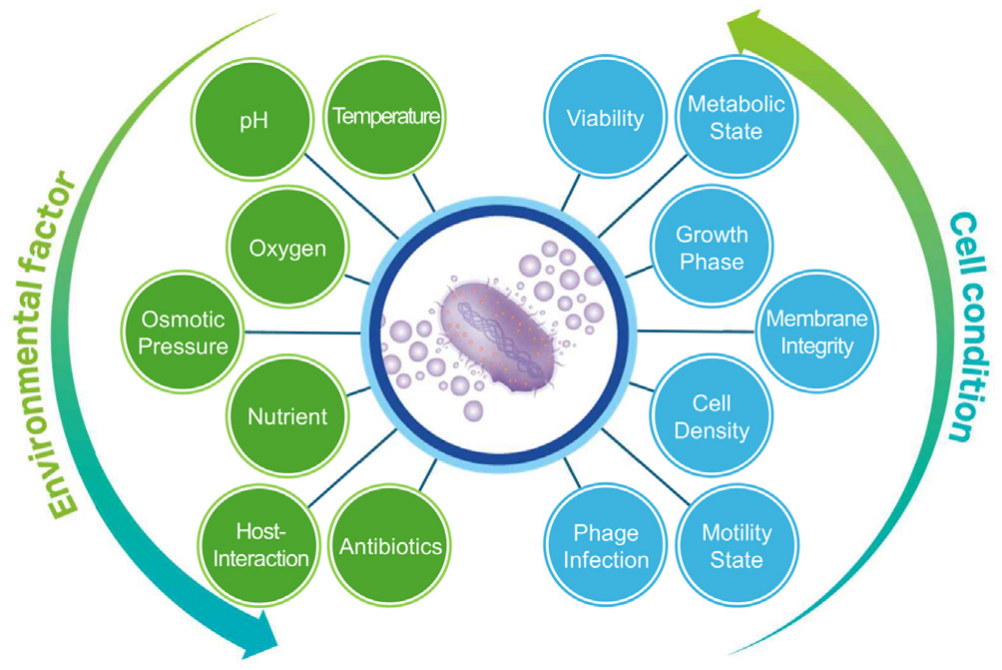
Environmental and cellular factors influencing bacterial EV production. Bacterial EV release is dynamically regulated by a variety of environmental factors (left), including temperature, pH, oxygen availability, nutrient conditions, antibiotics, osmotic pressure, and host interaction. Likewise, cellular states (right), such as viability, growth phase, cell density, phage infection status, metabolic activity, motility behavior, and membrane integrity, collectively influence the quantity and composition of secreted EVs. Understanding the interplay between these factors is essential for standardizing EV isolation and interpreting results across diverse microbial systems.

Bacterial growth conditions can affect the production, content, dead vesicles, yield, purity, and degree of potential contaminants, thus affecting the quality of EVs [19, 38]. Various factors, including nutrient availability, salt concentration, pH, and oxygen levels, can significantly impact the generation and functionality of EVs (Fig. 1)[25, 32, 38]. Unlike eukaryotic cells, bacteria usually show rapid proliferation with concurrent cell division and lysis that can complicate the isolation of intact EVs from live bacteria [39, 40]. Thus, a major challenge in EV purification is distinguishing genuine EVs from contaminants such as cell debris and death-associated particles. It is quite difficult to discriminate natural EVs from other particles from dead bacteria. Thus, minimizing bacterial death with careful maintenance of viability and optimization of growth conditions tailored to each species is critical. Importantly, bacterial growth dynamics vary depending on species and environmental conditions. For this reason, it is highly recommended to establish a growth curve for each strain to fine-tune culture parameters and obtain an optimal growth phase. For experimental reproducibility, batch culture techniques are widely used for consistent bacterial growth, with aliquoted bacterial stocks stored at -80°C for providing long-term preservation and consistency across experiments to prevent genetic mutation [41]. For large-scale bacterial cultures, performing serial dilutions for sub-culture is effective in achieving an optimal optical density (OD) [19]. Inoculating large-scale cultures directly from frozen stocks can cause inconsistent bacterial growth from batch-to-batch. Thus, initiating bacterial culture with a small-volume starter culture (3-5 ml) enables stable bacterial growth. This dilution-based adaptation strategy helps synchronize bacterial populations and stabilize bacterial growth within a short time frame [42].

### Bacterial Growth Phase

The bacterial growth phase critically shapes EV properties. During the logarithmic (log) phase, bacteria undergo rapid growth with heightened metabolic activity, resulting in extensive EV production [43]. However, the stationary phase causes stress responses and quorum sensing of bacteria, which can modify EV contents with accumulation of EVs produced at earlier phases and generation of vesicles from bacterial lysis [44, 45]. To reduce these artifacts, EVs are often harvested during the mid-to-late log phase, in which bacterial viability remains high with minimal substantial cell death (Table 1). For instance, *Escherichia coli* EVs are commonly collected before reaching an OD_600_ of 1.0 corresponding to the late-log phase to reduce contamination from dying cells [46]. Recent studies have demonstrated distinct characteristics of growth phase-specific EVs. For example, *Helicobacter pylori* EVs isolated from the late-exponential phase contain higher levels of protein, DNA, and RNA than EVs isolated from early-exponential and stationary growth phases [47, 48]. Similarly, Gram-positive *Staphylococcus aureus* EVs exhibit distinctive cargo profiles across growth phases, with variations in metabolite and protein compositions [49]. *Bacillus subtilis* EVs also display differential protein expression patterns between sporulation and vegetative growth phases, reflecting bacterial developmental transitions [50]. Along with changes in EV compositions, the bacterial growth phase can influence EV functionality. For example, *Streptococcus pneumoniae* EVs interact differently with macrophages depending on their harvest point [51]. Additionally, EVs from *Lacticaseibacillus rhamnosus* harvested during the late stationary-phase (48 h) exhibit higher immunomodulatory capacity than those collected at earlier time points (6 h and 12 h) [38]. This enhancement is attributed to elevated levels of lipoteichoic acid (LTA), a key TLR2 activator essential for immune modulation [38]. Although *L. rhamnosus* EVs derived from the stationary-phase exhibit enhanced therapeutic potential, some of them might have originated from cell death of bacteria rather than active secretion. These studies provide insight into bacterial EV functionality, which is largely affected by parental bacterial growth status and density. Thus, careful evaluation of their growth is needed. Likewise, determining the optimal timing for EV harvesting is crucial to acquire natural purity-favoring log-phase EVs for higher yields and potential functional advantages. Further studies are required to distinguish naturally secreted EVs from death-associated vesicles in late stationary-phase cultures to understand bacterial EV-mediated intercellular networks.

### Culture Media Composition

Next, the composition of growth media serves as another critical factor influencing bacterial EV release and content. Complex media such as lysogeny broth (LB), brain heart infusion (BHI), and tryptic soy broth (TSB) contain rich nutrients and support robust bacterial growth, making them suitable for large-scale EV production [52-54]. It has been reported that 2-fold concentrated LB can induce 70% increase of EV production in *Vibrio vulnificus* compared to standard LB [25]. However, complex media contain undefined components from yeast or tissue extracts that might interfere with downstream applications. Although autoclaving can disrupt vesicle-like artifacts and denature proteins, residual impurities may still persist [55]. For this reason, defined media are used in EV isolation because they could allow precise control over nutrient availability and bacterial metabolic states while minimizing unwanted impurities in undefined media. Moreover, adding specific supplements to defined media can significantly alter the quantity and molecular profiles of EVs. For example, supplementing 1% glycine to *E. coli* Nissle cultures led to a 69-fold increase in EV protein content and a 51-fold increase in lipid content while simultaneously reducing lipopolysaccharide (LPS) endotoxin activity [56]. This precisely defined culture medium ensures greater consistency in EV production and composition, providing advantages for certain targeted applications that demand strict control over purity and molecular content, such as for vaccine development [57, 58]. Nonetheless, the limited nutrient availability in defined media often results in stress and slower growth of bacteria, which can affect EV secretion kinetics [19]. Therefore, the choice of bacterial culture medium is important for robust bacterial growth and EV release, especially for large-scale EV production.

### Environmental Factors

Environmental factors are key determinants of bacterial EV release (Fig. 1) [59]. Temperature is a major effector of bacterial growth. Many bacterial species show optimal growth and EV release at around 37°C. However, their production varies according to the temperature across species. For instance, *E. coli* increases EV production at elevated temperatures, whereas *Pseudomonas aeruginosa* shows little changes in EV production between 25°C and 37-39°C [59, 60]. Similarly, *V. vulnificus* usually grows optimally at 30°C, but a higher temperature at 37°C induces a marked increase of EVs [25]. pH is another crucial factor affecting bacterial metabolism and EV properties [61]. Although many bacterial species favor a neutral pH (7.0-7.4) for optimal growth and EV production, alteration in pH can affect EV formation. For example, the facultative intracellular pathogen *Salmonella Typhimurium* increases EV production inside acidic vacuoles of host cells by changing expression of outer membrane proteins [62]. Notably, oxygen availability affects bacterial EV production. Some facultative anaerobes benefit from proper oxygenation and agitation (*e.g.*, shaking) for promoting consistent growth and EV release [63]. In contrast, obligate anaerobes such as gut microbiota require strictly anaerobic conditions, often necessitating anaerobic chambers to sustain their viability and EV release [64, 65]. Although opportunistic strains including *P. aeruginosa* and *S. aureus* exhibit flexibility in oxygen tolerance, they still require careful oxygen adjustments to optimize EV release [66]. Salt concentration in culture medium also affects bacterial viability and EV production [67, 68]. Proper osmotic conditions during bacterial growth can support consistent EV formation, whereas extreme hypertonic or hypotonic conditions can induce osmotic stress known to disrupt EV membranes [68]. Finally, specific stressors such as antibiotics can modulate the yield and composition of EVs [69]. For instance, treatments with polymyxin B or D-cycloserine can enhance EV release potentially as part of a bacterial defense mechanism [59].

Overall, optimizing environmental conditions for bacterial growth while avoiding excessive stress to parental cells is essential for obtaining EVs with high purity and yield. Establishing species-specific culture conditions allows precise control over EV production and provides valuable insights into how environmental factors mimic natural infection sites or host-associated niches, such as pH fluctuations in inflamed tissues, hypoxic conditions in the gut, and mucin-rich environments in gastrointestinal tracts. Understanding these adaptations of bacteria with EV release can facilitate meaningful comparisons between *in vivo* bacterial EVs and those produced *in vitro* under laboratory conditions. To enhance reproducibility and standardization in EV research, detailed documentation of bacterial growth parameters, including culture conditions and OD thresholds, should be thoroughly reported in experimental protocols [17].

## Removal of Bacteria and Harvesting Supernatant

**Fig. 2:**
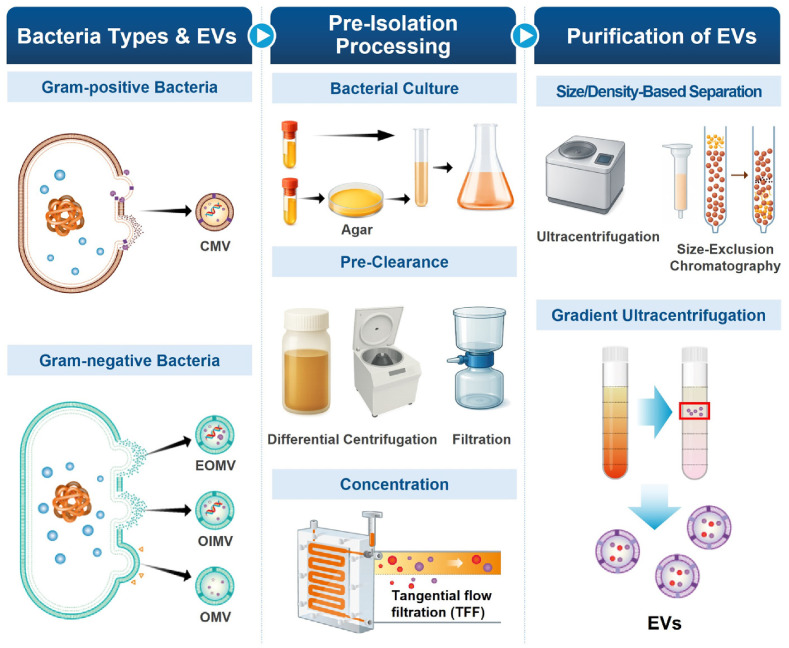
Overview of bacterial EV isolation workflow. Different types of EVs are produced by Gram-positive (*e.g.*, CMV) and Gram-negative bacteria (*e.g.*, OMV, OIMV, EOMV). EV isolation begins with pre-isolation processing including bacterial culture, pre-clearance of culture supernatant, and concentration. For EV purification, size- or density-based separation methods such as size-exclusion chromatography and ultracentrifugation are used. Density gradient ultracentrifugation enables high-purity EV fractionation based on EV density.

Once bacterial growth is optimized, the next critical step is obtaining a bacteria-free supernatant to prevent contamination from live bacteria, cell debris, and nucleic acid-rich substances (Fig. 2), which can lead to EV aggregation and loss (EVs here refer collectively to CMVs, EOMVs, OIMVs, and OMVs) [70, 71]. Therefore, complete removal of bacteria is fundamental to EV isolation, as residual cells and their byproducts can largely affect EV purity and functionality [17]. To reduce contamination, bacteria should be removed immediately after harvesting them in conditioned medium at an appropriate OD or growth phase. Delayed processing and prolonged exposure to room temperature can allow bacteria to continue growing. Sudden temperature shifts to ice can also induce stress or lysis of bacteria [72]. This is particularly concerning for anaerobic bacteria as oxygen exposure can trigger lysis to release sticky materials such as nucleic acids, which can hinder bacterial pelleting while promoting EV aggregation [73-75]. Moreover, freezing bacterial culture medium before bacterial removal is strongly discouraged because freeze-thaw cycles are known to rupture bacteria and induce further contamination of bacterial debris with ruptured cytoplasmic contents.

### Differential Centrifugation of Bacteria Cultures

Differential centrifugation (pre-clearing phase) is widely used to remove bacteria and their debris. The success of this technique largely depends on the precise selection of centrifugal forces in a step-wise manner to ensure efficient bacteria removal while minimizing EV loss and damage. This stage requires a carefully chosen centrifugal force to minimize shear stress, which could otherwise lead to bacterial lysis and the release of unwanted cellular components, causing a low EV purity [76]. Since many bacterial cells typically range from 1-3 μm in diameter, low-speed centrifugation (*e.g.*, 3,000-6,000 ×*g*) is applied to effectively remove intact bacterial cells [46, 77]. For representative laboratory bacteria *E. coli*, a relative centrifugal force (RCF) of 6,000 ×*g* for 20 min at 4°C is commonly used to sediment intact bacterial cells (Table 1). This step is recommended to be repeated at least twice to ensure complete removal [78]. However, some bacterial species present additional challenges. For example, for *A. muciniphila*, which grows in mucin-rich media, it is difficult to completely remove them due to their adhesive properties to mucin, which can cause inefficient pelleting [12]. In addition, bacteria with low viability (*e.g.*, some obligate anaerobes, slow-growing bacteria) are prone to lysis during centrifuge, potentially leading to contamination with cellular debris and nucleic acids in the supernatant [79]. For these bacteria, repeating the low-speed centrifugation step can improve bacterial removal efficiency with while preventing their lysis before proceeding to the next purification step. To check bacterial clearance, plating the supernatant onto an agar plate can be used to confirm the absence of viable bacteria by colony counting.

Once bacterial cells are removed, the supernatant is subjected to higher-speed centrifugation to eliminate smaller cell debris and large aggregates [80]. Typically, centrifugation at 10,000 ×*g* for 20 min is sufficient to pellet non-EV particles from the supernatant (Table 1) [81, 82]. This step can also be repeated, if necessary, to improve the removal of residual contaminants. Careful transfer of the supernatant is important, as disturbing the pellet may reintroduce debris into the purified fraction. It is also recommended to avoid collecting the entire supernatant down to the last drop, as this can carry over unwanted particles into subsequent steps.

Differential sequential centrifugation steps take advantage of differences in size and mass among bacterial cells, cellular debris, and EVs, enabling an effective pre-purification process [83, 84]. Differential centrifugation provides an indispensable, cost-effective, and scalable initial step in EV isolation [76]. However, differential centrifugation alone does not achieve absolute size-dependent separation, as sedimentation is also affected by particle density and cargo composition [85].

### Filtration of Supernatant

Microfiltration (polishing phase) is commonly used to completely remove live bacteria for obtaining sterile bacterial EVs [86]. Filtration is a crucial step for EV purification as it can remove host debris, large particles, and potential contaminants. Filter pore size plays a key role in determining the efficiency of bacterial and debris removal. Typically, 0.22 μm or 0.45 μm pore-sized filters are used to eliminate bacteria while allowing EVs to pass through because most EVs have sizes ranging from 20 nm to 150 nm (Table 1). Filters with pore sizes of 0.22 μm and 0.45 μm can retain particles larger than 220 nm and 450 nm, respectively (±20%) [7]. In some cases, a sequential filtration approach using both 0.45 μm and 0.22 μm filters can be applied for more stringent bacterial removal [87]. The initial larger pore filter helps eliminate lipid-rich or sticky components that could otherwise clog the finer 0.22 μm filter, thereby reducing consumption of expensive disposable filters. However, filtration is not infallible despite its utility. Certain bacterial species have been shown to pass through 0.22 μm-pore sized filters and grow thereafter [88]. Therefore, parallel validation by microbial plating after filtration is necessary to confirm the absence of viable bacteria [89]. Filter pore sizes ranging from 0.45 μm to 0.1 μm can be selected to improve sample quality without compromising EV recovery depending on the bacterial strain and culture conditions.

Another technical consideration is the aggregation of EVs during filtration. This aggregation could be induced by the lipid-rich nature of EV membrane and its interaction with residual proteins or nucleic acids [90]. Although filtration can assist in removing aggregates, excessive filtering pressure and repetitive membrane usage may result in EV loss or membrane fouling. To mitigate such risks, temperature control is crucial to maintain supernatants at 4°C or on ice immediately after bacterial removal. Freezing the supernatant is not recommended as freeze-thaw cycles can alter media compositions and disrupt EV integrity [91]. If immediate processing is not feasible, storing the supernatant at 4°C for overnight is generally preferable rather than freezing. Additionally, to improve EV recovery, gentle swirling or agitation during filtration can help prevent vesicle adhesion to the filter membrane. The choice of filter material can also influence sample retention. Hydrophilized polyvinylidene difluoride (PVDF) membranes are recommended due to their low protein-binding properties, which can reduce non-specific binding of EVs on the membrane [92]. Finally, the use of stabilizing or cryo-protective agents such as trehalose, polyethylene glycol, and naturally derived polysaccharides has been found to be able to reduce EV aggregation in supernatant [93, 94]. In particular, trehalose has been shown to be able to enhance colloidal stability and improve particle yield by minimizing vesicle-surface interactions in human EV studies [93]. Examining whether such additives can similarly benefit bacterial EVs as observed in human EVs would be useful.

In summary, proper centrifugation and filtration strategies are required according to specific bacterial species and culture conditions to improve EV yield, quality, and reproducibility. Selecting appropriate centrifugal forces, choosing suitable filter pore size, minimizing membrane fouling, and considering strain-specific variables will allow researchers to confidently isolate bacterial EVs for functional studies and multi-omics analyses.

## Concentration of Supernatant

Once a proper amount of pure supernatant (*i.e.*, free of bacteria and cell debris) has been harvested, concentrating the supernatant is often required to obtain a sufficient EV yield. Reducing the sample volume can improve the efficiency of subsequent purification steps, with concentrating levels ranging from at least 2-fold to several hundred–fold, depending on experimental needs. Several methods are available for this purpose, each with distinct advantages and limitations.

### Ultrafiltration via Tangential Flow Filtration (TFF)

Ultrafiltration is a pressure-driven membrane filtration method widely used to concentrate EVs from culture supernatant [95]. Polyethersulfone or regenerated cellulose is widely used as ultrafiltration membranes. They offer high permeability and low fouling tendencies, enabling for the selectivity of diverse sized particles with pores in a range of molecular weight cut-offs (MWCO) from 10 kDa to 100 kDa and allowing for precise separation of EVs from smaller contaminants [96]. Traditional ultrafiltration systems rely on normal dead-end flow filtration, where the fluid moves perpendicular to the membrane surface. However, this approach has limitations due to membrane clogging, as filtered particulates will accumulate on the membrane to form a ‘filter cake’ that can reduce flow efficiency and cause fouling over time [97]. By contrast, TFF has become a frequently used method for EV concentration because it can efficiently concentrate large sample volumes and prevent membrane fouling (Fig. 2) [98]. Unlike conventional filtration where particles accumulate on the membrane, TFF directs liquid tangentially along the membrane surface, generating shear forces that can block clogging and allow continuous filtration [97]. This method is particularly suitable for concentrating bacterial culture supernatants. It supports reproducible, scalable large-scale EV production as in eukaryotic EVs [99]. Earlier systems such as the QuixStand benchtop equipment have been employed for supernatant concentration and diafiltration [78]. The QuixStand system was practical for laboratory-scale applications due to its compact size and adaptability. However, this unit required manual pressure adjustments to prevent membrane damage, making it less useful for high-throughput workflows. Due to these limitations, this system has been discontinued and more advanced TFF platforms have since been developed [80]. Among these, the AKTA flux (Cytiva) is a versatile crossflow filtration system that uses hollow fiber cartridges and programmable settings suitable for diverse experimental scales [100]. Currently, different from hollow fiber systems, modern TFF workflows often adopt cassette (flat-sheet) membranes as they offer high packing density and pump compatibility, enabling greater throughput, more efficient large-scale bioprocessing, and higher yield of EV isolation [101]. In addition, alternative setups such as Pellicon 2 cassette filter membrane (Merck) combined with MasterFlex pump system (Cole-Parmer) can provide customizable configurations for EV concentration in flexible laboratory environments [102].

### Centrifugal Filter Units (CFU)

For small sample volumes, CFU systems (*e.g.*, Centriprep, Amicon, Vivaspin) offer a convenient and scalable alternative for concentrating EVs. Moreover, due to a dead volume in TFF systems, the final concentrated volume often remains around 100 ml, necessitating further concentration. To address this, CFU systems can be employed to achieve desired concentrations. CFUs operate by applying centrifugal force to drive liquid through a membrane with a defined MWCO, typically ranging from 10 kDa to 100 kDa, thereby retaining EVs while allowing smaller molecules to pass [103]. Sample concentration is usually performed using high-speed centrifugation (*e.g.*, 3,000 ×*g* for 10-30 min), with repeated cycles as needed depending on sample volume and filter specification. While CFUs offer simplicity and convenience, they might cause vesicle loss due to membrane clogging, particularly when samples are viscous or protein-rich [104, 105]. To minimize this, pre-filtration and cold processing (4°C) are recommended to reduce the consumption of expensive CFU filters. Despite these limitations, CFUs remain a valuable tool for small scale or pilot EV preparations, especially when they are used judiciously with proper membrane selection and volume control strategies [106].

### Precipitation-Based Methods

In addition to membrane-based methods, precipitation has been explored as an alternative method for concentrating EVs from culture supernatants [107-109]. Instead of physical concentration techniques, precipitation involves adding a high concentration of salts or polymers, which can disrupt hydrogen bonds and surface charges, leading to protein aggregation and facilitating EV isolation by centrifugation [95]. Ammonium sulfate is frequently used due to its high solubility and ionic strength, enabling bacterial EV precipitation through gradual addition with stirring at 4°C [95, 110]. It is considered a cost-effective and scalable process that does not require specialized equipment. However, a precipitation method has several drawbacks. Its use is limited with rich growth media, as various components can co-precipitate with EVs, restricting its applicability to minimal media. Furthermore, co-precipitation of bacterial components such as non-vesicular extracellular proteins can reduce sample purity and complicate downstream applications [111]. This method might also introduce variability in EV composition, as certain vesicle subtypes or aggregates may preferentially precipitate, potentially affecting reproducibility [112]. Additionally, residual precipitation agents must be thoroughly removed through extensive dialysis against a suitable buffer to prevent interference with subsequent analyses. Given these limitations, precipitation is generally not recommended as a standard method for bacterial EV purification. Similar to vesicles artificially generated through mechanical shearing, detergent treatment, or chemical induction, precipitated EVs might also exhibit structural and functional differences from naturally secreted EVs [113]. These artificially generated vesicles often with an aim to produce them in large quantities can lead to misinterpretation of EV characteristics and compromise the reproducibility of experimental applications. Therefore, precipitation methods are not suitable when high-purity or functionally intact EVs are required for downstream analyses.

In conclusion, ultrafiltration methods, particularly those based on TFF, are recommended. After ultrafiltration, a final concentrated supernatant can be sterilized with 0.22-μm pore membranes or high-speed centrifugation at 4°C to remove aggregates or precipitates that might have occurred during concentration [114]. Finally, after concentration, an additional filtration step (0.22-0.45 μm pore size) or high-speed centrifugation (*e.g.*, 10,000 ×*g* for 10-30 min) is recommended to remove aggregates prior to further purifications.

## Isolation and Separation of EVs

After concentrating a bacterial supernatant, further purification steps are essential to isolate pure nano-sized EVs. These steps focus on eliminating contaminants such as protein aggregates, cell debris, and bacterial appendages (*e.g.*, flagella, fimbriae, pili) while preserving the structural integrity and biological functionality of EVs. Popular techniques include ultracentrifugation, size-exclusion chromatography (SEC), ultrafiltration, and sucrose cushion, while it is difficult to apply immunoaffinity-based purification for bacterial EVs due to strain-level diversities [97]. Each method has distinct advantages and limitations in terms of purity, scalability, and compatibility.

### Ultracentrifugation

Ultracentrifugation is a conventional technique used to pellet EVs by applying high centrifugal forces, typically 100,000 ×*g* to 200,000 ×*g* for 1-3 h (Table 1). This method is relatively simple and fast. Although it is effective, it has several critical limitations that must be considered. Utilizing suboptimal RCF speed or insufficient spin time may result in incomplete EV recovery, leaving EVs in the concentrated supernatant and resulting in a low yield [84]. Conversely, excessive centrifugal force or prolonged duration can lead to heterogeneity in final preparations, such as a mixture of EVs, protein complexes, and other contaminants [84]. Moreover, high centrifugal forces can compact EVs into a dense pellet, leading to aggregation and structural damage, thereby reducing sample integrity and potentially altering biological functionality [115]. In human EV studies, ultracentrifugation has been associated with impaired functionality of EVs, potentially due to damaging forces exerted on vesicles during centrifugation at a high speed [76, 116]. Additionally, contamination risk is increased due to co-pelleting of cellular debris or bacterial appendages, which share similar sedimentation properties with EVs. Furthermore, ultracentrifugation may lead to structural deformation or rupture of EVs. Shear stress during high-speed spinning can destabilize vesicle membranes, compromising their integrity. Moreover, EV pellets often exhibit viscous or adhesive properties due to their lipid and nucleic acid contents, making them difficult to harvest and thereby increasing the risk of sample loss. Nevertheless, ultracentrifugation remains the most commonly used method due to its cost-effectiveness, simplicity, and accessibility, although careful consideration is required for optimal yield and purity.

### Size-Exclusion Chromatography

SEC is a gentle and effective method for isolating EVs based on size differences [117, 118]. In this technique, EVs pass through a column filled with porous beads, where larger particles will be eluted first, while smaller contaminants (*e.g.*, proteins, metabolites, solutes) are retained longer within pores [76]. Traditionally, SEC requires large fast performance liquid chromatography systems, limiting its practicality for routine EV isolation in typical laboratory settings [100]. However, commercially available pre-packed SEC kits such as qEV (Izon) have significantly simplified EV purification by utilizing passive gravity-driven flow, which can minimize vesicle aggregation and preserve structural integrity [97, 119]. These ready-made columns enable rapid purification with minimal (< 5%) cellular protein contamination in a human EV study [120]. To optimize the purity and yield, the pore size of SEC beads should be carefully considered as it determines the resolution for separating EVs from non-EV components with different sizes. Selecting an appropriate pore size is critical to ensure efficient exclusion of larger contaminants while allowing precise fractionation of target EV population [121]. Prior to loading a concentrated bacterial supernatant, the SEC column should be washed with PBS to remove trace amounts of storage materials (*e.g.*, glycerin) to activate the separation matrix [117]. EVs can subsequently be eluted using 0.1 μm-filtered PBS [122]. SEC fractionation follows standardized elution profiles. EVs are typically eluted in specific early fractions, thereby ensuring reproducibility and consistency across experiments [122]. This predictable fractionation pattern allows researchers to collect EV-rich fractions while discarding later fractions containing smaller contaminants such as free proteins and metabolites [100]. As a result, SEC has been widely adopted as a standalone method or in combination with ultracentrifugation or density-gradient centrifugation to enhance EV purity (Fig. 2). It is particularly well-suited for being integrated into analytical pipelines involving proteomics and genomics [114, 123]. Its limitations include high cost, maintenance requirements including membrane washing and proper storage, and limited scalability for high-throughput or clinical practice.

### Alternative Methods

Recently, the use of superabsorbent polymer beads has been explored as an alternative method for concentrating human EVs from cell culture medium [117, 124]. These beads composed of polyacrylamide-to-itaconic acid can selectively absorb liquid (water, media) while retaining EVs in a significantly concentrated state [124]. Although this method has not been applied to isolate bacterial EVs, it might be a promising technique for isolating host-derived fluids such as human urine or for large-volume processing. It offers improved isolation performance over conventional filtration [125]. By using concentrated EVs in supernatants, this method does not require further centrifugation steps. In addition, its EV losses are low. However, smaller byproducts such as metabolites and soluble proteins might still be retained, depending on their interactions with the polymer surface.

In some cases, a “cushion” of the concentrated supernatant is placed atop a dense medium at the bottom of the ultracentrifuge tube to form a step gradient [126]. This approach is useful for enhancing part of the separation by making it easier to re-suspend EVs and prevent damage to vesicles that might not withstand pelleting. To mitigate these risks, rather than stacking EVs by ultracentrifugation, a sucrose or iodixanol density cushion can be employed to absorb impact forces. As the word “cushion” implies, this approach can prevent vesicles from undergoing excessive mechanical stress by allowing them to form a distinct band at the interface of the sucrose or iodixanol layer [126]. This method not only minimizes contamination of soluble proteins and protects EVs from shear-induced damage, but also facilitates easier EV retrieval compared to direct ultracentrifugation. However, residual sucrose or iodixanol might remain in the sample. Thus, additional washing steps are needed for accurate characterization or quantification of EVs.

Many studies choose to terminate EV purification at this stage, particularly when large quantities are required, such as for *in vivo* mouse experiments. If QC methods, including transmission electron microscopy (TEM) and nanoparticle tracking analysis (NTA), confirm that the purity is sufficient, further purification can be omitted. Nonetheless, researchers working on immune response studies must be cautious as contamination with fimbriae or pili could affect immunological outcomes [1]. While EVs purified up to this step can achieve reasonable purity for many bacterial strains [64, 67], additional purification is strongly recommended for high-resolution analyses such as –omics studies to ensure the highest sample purity and consistency [8, 46]. This is particularly important when preparing EVs under conditions compatible with good manufacturing practice (GMP) for translational applications [127, 128].

## Density Gradient Centrifugation

Density gradient centrifugation is one of the most effective methods for further purifying bacterial EVs after initial isolation steps (Fig. 2) [129]. By leveraging buoyant density differences, this method can effectively separate EVs from soluble proteins, free genetic materials, and other contaminants, providing high-purity EV compatible with follow-up analyses [130].

In earlier studies, sucrose gradients were often used for density-based EV separation due to their cost-effectiveness and buoyant density-based fractionation [131]. However, they require longer centrifugation time (> 16-20 h) because of their high viscosity [46, 132]. Furthermore, manual preparation can introduce variability in sucrose concentration, as minor inconsistencies in dissolution can affect density uniformity between experiments [130]. Moreover, sucrose aggregates may form during dissolution and prolonged storage, even under refrigeration, might increase the risk of microbial contamination. Compared to sucrose gradients, OptiPrep (iodixanol-based) gradients provide a more precise and reproducible approach for EV purification [133]. Iodixanol can form isosmotic solutions at all densities, preserve EV integrity, minimize aggregation, and ensure high-purity vesicle recovery [131]. It can also improve the separation of heterogeneous EV populations while avoiding density shifts observed in sucrose gradients during storage. Note that commercially available (ready-made) OptiPrep stock solution (Sigma-Aldrich) is supplied at 60% (w/v), not 100%, with a density of 1.320 ± 0.001 g/ml [134]. Therefore, precise dilution calculations are essential for gradient preparation. The working solution (50%) should be prepared first using a diluent buffer (Table 2) instead of PBS, as the use of PBS may lead to variations in salt concentration across gradient fractions [135]. All dilution solutions should be filtered with 0.45 μm membrane filters before use. A standard iodixanol gradient typically ranges from 0% to 50% (v/v), although specific concentrations should be optimized based on bacterial strain and EV density [131].

Briefly, bacterial EVs are resuspended in an OptiPrep working solution to adjust concentration to 40-50% at the bottom, followed by sequential layering of lower concentrations (5-30%) (Table 2). The gradient is then centrifuged at 100,000 ×*g* to 200,000 ×*g* at 4°C for 2-3 h using an ultracentrifuge [8, 78]. Optimizing centrifugation parameters (*e.g.*, force, time, and rotor type) for specific bacterial strains and EV density is crucial for reproducibility. While some protocols use longer centrifugation time (up to 16-20 h) to generate a linear continuous gradient, excessive spins may lead to multiple bands due to vesicle heterogeneity [136]. Continuous gradients allow finer separation of EV subpopulations. However, selecting the appropriate fraction requires extensive QC and validation. For efficient EV recovery, step gradients are preferred over continuous gradients, as they can concentrate EVs into distinct bands, thus minimizing vesicle loss (Table 1). If the goal is to isolate a homogenous vesicle subtype, flow cytometry such as CytoFLEX (Beckman) which can dissolve nano-sized vesicles based on their size and antigen expression might be a more suitable alternative [137, 138].

After centrifugation, fractions are collected from the top and analyzed for EV presence. Fraction collection should be tailored to EV characteristics and intended application. Strategies such as combining several fractions or adjusting the step gradient percentage can improve yield and purity. If EV amounts are sufficient, a visible band at the interface (typically at 1.1-1.24 g/ml, although it is strain-dependent) can be harvested directly [139, 140]. Otherwise, EV presence should be traced using density measurements, protein quantification, or further QC.

While density gradient centrifugation can significantly enhance EV purity, it does not fully eliminate protein complexes or bacterial components with similar densities. Frequently observed contaminants in EV proteomics studies include ribosomes, multi-tRNA synthetases, and heat-shock proteins [141]. These entities may co-purify with EV due to their overlapping buoyant densities, complex formation, or high abundance in bacterial cultures rather than representing genuine EV components [142]. Accordingly, to achieve the maximum purity and reproducibility for EV preparations, combining ultrafiltration and SEC with density gradient centrifugation is highly recommended [143]. This integrated approach can effectively remove aggregated vesicles and unwanted components, thus ensuring greater consistency in EV isolations.

## QC and Storage of Purified EVs

For reproducible EV experiments, proper QC and storage methods are critical. Formally, purified EVs are characterized using TEM and NTA to confirm their nanoscale size and purity. However, unlike human EVs, bacterial EV lack universal markers such as CD63 and CD81, which are commonly used for human EV validation [144]. Consequently, bacterial EV studies tend to prioritize passing QC criteria, such as meeting size and purity thresholds, rather than ensuring consistency in methodology due to species diversity, contamination risks, and scalability issues [1]. This discrepancy highlights an urgent need for bacterial EV-specific reporting standards. Efforts akin to MISEV guidelines for eukaryotic EVs are essential.

A key challenge in bacterial EV isolation is the high cost of immunoaffinity-based methods, which can further complicate QC consistency (Table 3). While defining common molecular signatures including PAMPs such as Gram-positive LTA and Gram-negative LPS can help identify EVs, these PAMPs can also label contaminants from cell debris or aggregates, thus limiting their reliability as EV-specific markers [145, 146]. Instead, outer membrane proteins and surface-exposed proteins provide more specific validation targets. For example, an antibody against outer membrane protein A (OmpA) of *E. coli* or *Acinetobacter baumannii* is commercially available. It can be used to confirm EV QC via Western blotting or gold-labeling TEM [147, 148]. Similarly, *S. aureus* EVs contain IgG-binding protein (SbI), which can be easily detected using anti-IgG-gold labeling, thus offering a reliable validation marker [8]. Nevertheless, certain proteins (*e.g.*, GroEL or flagellin) are inconsistently classified as either EV-enriched or EV-depleted, underlining the context-dependent nature of EV markers [139]. Although such protein-based markers are not universal, some microbial epitopes are shared among certain bacterial groups, which can facilitate bacterial EV identification while helping researchers distinguish them from non-vesicular contaminants and host-derived EVs in biological samples.

Quantification and normalization strategies are also important. Protein concentration (*e.g.*, BCA assay), lipid content, and vesicle counts with NTA, dynamic light scattering, or flow cytometry are commonly employed for standardization [149]. Depending on the study objective, different approaches can be used to compare total EV counts between samples. SDS-PAGE with Coomassie Brilliant Blue or silver staining can be used to assess protein pattern differences between whole cells and EV fractions, thus providing additional QC measures [46, 150].

As a final consideration, proper storage of bacterial EVs ensures reliable experimental outcomes. EVs are inherently sensitive to environmental changes due to their phospholipid bilayer structure and cargo of diverse biomolecules [151]. The MISEV2023 guidelines for eukaryotic EV storage strategies might also be applicable to bacterial EVs, as they emphasize standardized conditions for preserving vesicle integrity, functionality, and biomolecular content [1]. However, further validation is needed to confirm their suitability given that eukaryotic and bacterial EVs show structural and compositional differences. To preserve EV integrity and functionality while avoiding damage, aliquoting EV samples before storage can minimize freeze-thaw cycles. Numerous studies have demonstrated that repeated freeze-thaw events can lead to significant degradation of EVs, including reduction in particle number, increase of aggregation, and loss of bioactivity [149, 152, 153]. Therefore, EVs should be ideally stored at -80°C in single-use aliquots. Rapid freezing techniques such as immersion in dry ice or liquid nitrogen immediately after EV isolation can help preserve their properties. Alternatively, freeze-drying (lyophilization) has emerged as a potential strategy for enhancing long-term stability and transportability of EVs [91]. Recent studies have shown that freeze-drying with appropriate cryo- and lyoprotectants such as disaccharides (*e.g.*, trehalose) combined with dextran or glycine can successfully maintain the physicochemical integrity of human EVs [154, 155]. Furthermore, biological excipients such as EV-depleted serum and secretome can enhance EV preservation and cellular uptake, indicating their dual roles as stabilizers and functional enhancers [91, 156]. Based on these promising results, the impact of lyophilization on functional activity of bacterial EVs should be explored post-reconstitution, particularly regarding immune modulation and host-pathogen interaction.

Ultimately, regardless of the chosen preservation method, minimizing freeze-thaw cycles at the earliest stage of EV handling is essential. By incorporating standardized QC metrics and optimized storage protocols, bacterial EV research can achieve greater reproducibility, ensuring high-quality EV for biological relevance, including functional assays and molecular characterization.

## Concluding Remarks

**Fig. 3:**
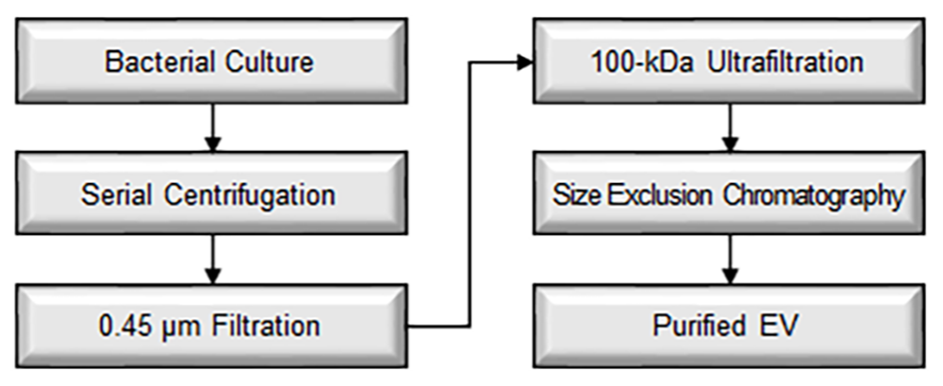
Minimal-optimal suggested protocol for bacterial EV isolation. Stepwise workflow integrating minimal essential methods with optimal components. Designed to support reproducible and scalable EV purification across diverse laboratory settings.

Over the past few decades, the bacterial EV field has made remarkable strides, yet a standard EV isolation protocol remains a major challenge. Unlike human EVs, bacterial EVs rely heavily on physical separation rather than biochemical approaches due to species diversity and the absence of universal microbial markers. To ensure reproducibility, establishing minimal yet effective purification guidelines is essential. As a step toward future standardization, we propose a unified and practical EV isolation workflow with minimal equipment requirements (Fig. 3, Table 4). Such streamlined guidelines will support reproducibility across varying laboratory settings and help bridge resource gaps in bacterial EV research.

Beyond *in vitro* isolation, distinguishing bacterial EVs from host biological fluids, stools, and tissues remains a hurdle as they share overlapping physicochemical properties [1, 4]. Refining strategies for accurate differentiation will provide clearer insights for clinical studies, particularly for microbiome research [157-159]. Leveraging insights from human EV studies, such as single-vesicle profiling (*e.g.*, sequencing-based approaches), can help us define the heterogeneity of bacterial EV subtypes and improve differentiation strategies [160]. Improving bacterial culture conditions and optimizing isolation methods will enhance EV quality and consistency, which in turn can unlock their therapeutic potential as non-replicative yet immune-modulatory agents to enhance clinical safety [38]. Their promise as vaccine platforms and diagnostic biomarkers further reinforces the importance of establishing robust purification standards.

## Figures and Tables

**Table 1:** Reported methods for EV purification from representative bacterial species.

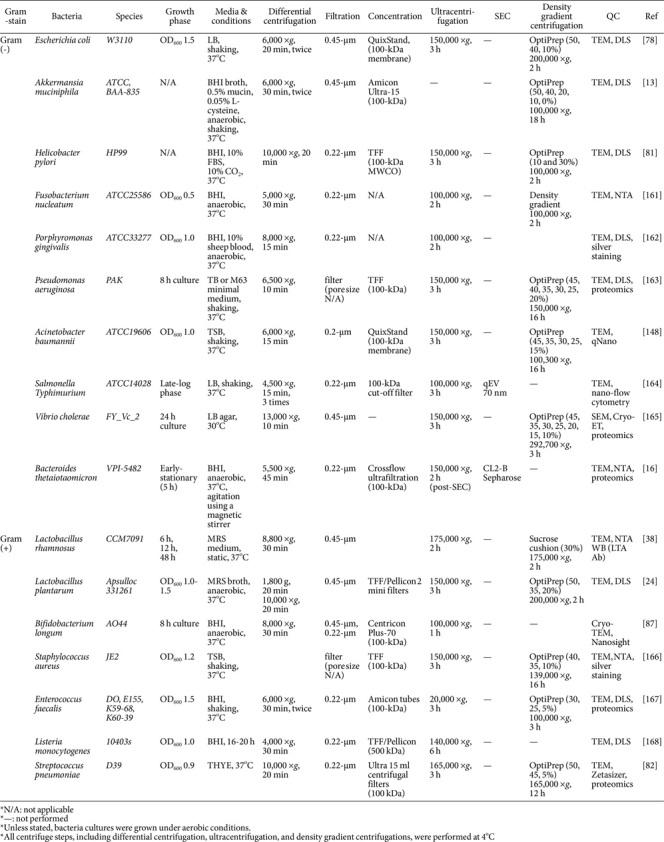

**Table 2:** Preparation of a stepwise Iodixanol (OptiPrep) gradient (v/v).

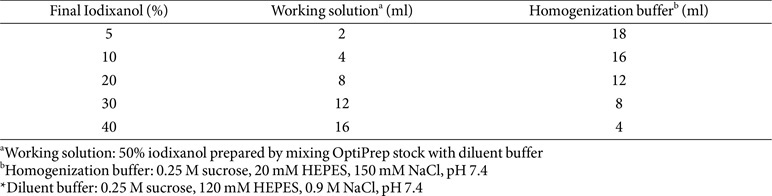

**Table 3:** Representative bacterial EV markers and their validation methods.

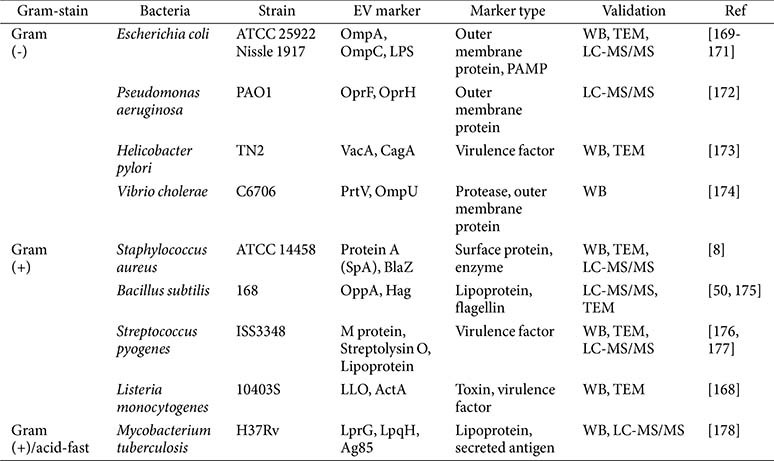

**Table 4:** Minimal equipment and materials required for each step of bacterial EV purification.